# Effectiveness of S-PRG–based varnish and bioactive glass–based air polishing in the management of dentin hypersensitivity and their effects on tooth color: a 12-month randomized clinical trial

**DOI:** 10.1186/s12903-026-09283-6

**Published:** 2026-07-20

**Authors:** Omnia M. Sami, Dina Kamal, Essam A. Naguib, Asmaa Aly Mohamed Yassen

**Affiliations:** 1 Department of Conservative Dentistry, Faculty of Dentistry, Cairo University, Cairo, Egypt; 2 Department of Conservative Dentistry, Faculty of Oral and Dental Medicine, Future University in Egypt, Cairo, Egypt; 3 Department of Clinical Dental Sciences, Faculty of Dentistry, Ibn Sina University for Medical Sciences, Amman, 16197 Jordan

**Keywords:** Bioactive materials, Dentin hypersensitivity, Dentin tubule occlusion, Randomized clinical trial, Visual Analog Scale

## Abstract

**Objectives:**

To evaluate and compare the 12-month clinical effectiveness of a surface pre-reacted glass-ionomer (S-PRG)–based resin varnish and a bioactive glass–based air-polishing system in the management of dentin hypersensitivity and their effects on tooth color.

**Methods:**

This randomized clinical trial included 34 participants diagnosed with dentin hypersensitivity. Participants were randomly allocated (1:1) to receive either an S-PRG–based varnish or a bioactive glass–based air-polishing treatment. The primary outcome was pain intensity measured using the Visual Analog Scale (VAS), while the secondary outcome was tooth color change. Pain intensity was assessed in response to tactile, evaporative, and thermal stimuli using a 0–10 VAS. Tooth color change was evaluated using modified United States Public Health Service (USPHS) criteria. Assessments were performed at baseline, immediately post-treatment, and after 1, 6, and 12 months. Parametric VAS data were analyzed using independent t-tests for intergroup comparisons and repeated-measures ANOVA for intragroup comparisons. Categorical data were analyzed using the Mann–Whitney U test for intergroup comparisons and the Friedman test for intragroup comparisons. Statistical significance was set at *P* ≤ 0.05.

**Results:**

The S-PRG–based resin varnish group demonstrated significantly lower VAS scores than the bioactive glass-based air-polishing system group immediately after treatment and throughout follow-up (*P* < 0.05). At 12 months, the bioactive glass–based air-polishing system group showed a significant relapse in pain scores, whereas the S-PRG–based resin varnish group maintained sustained pain reduction across all stimuli (*P* < 0.001). Immediately after treatment, the S-PRG–based resin varnish group exhibited temporary tooth color change, while no immediate color alteration was observed in the bioactive glass–based air polishing group. No significant differences in tooth color were seen between groups at 1, 6, or 12 months.

**Conclusions:**

S-PRG–based resin varnish demonstrated greater sustained reduction in dentin hypersensitivity over the 12-month follow-up period compared with the bioactive glass–based air-polishing system and may represent a promising option for moderate-term symptom control.

**Trial registration:**

https://clinicaltrials.gov/, NCT05981625, Registered 31 July 2023.

## Introduction

Dentin hypersensitivity (DH) poses a significant clinical challenge for both dental practitioners and patients. For patients, it is distressing and disrupts daily life. For clinicians, management is difficult due to the limited long-term effectiveness of most desensitizing agents and insufficient evidence for a single best treatment approach [1].

A systematic review and meta-analysis published in 2019 reported that the global prevalence of dentin hypersensitivity ranges widely from 4.8% to 62.3%. Young adults, particularly those aged 18–44, have a high prevalence of approximately 43.9%, highlighting the clinical importance of this condition in the adult population [2, 3].

Loss of enamel or cementum exposes dentin, making the tooth sensitive to thermal, tactile, osmotic, and evaporative stimuli. Clinically, dentin hypersensitivity is defined by a sudden, sharp, and short-lasting pain response [4]. Several theories have been proposed to explain the mechanism of this pain. However, the hydrodynamic theory is the most widely accepted and scientifically supported [5]. According to this theory, external stimuli induce fluid movement within open dentinal tubules. This mechanically stimulates nerve endings at the pulp–dentin complex, producing pain. Removal of the smear layer increases dentin permeability and intensifies tubular fluid movement, worsening the hypersensitivity. Therefore, most therapeutic strategies for DH aim either to reduce nerve excitability or to occlude dentinal tubules to limit fluid flow and reduce pain [6].

Recently, a light-cured, resin-based varnish with surface pre-reacted glass-ionomer (S-PRG) fillers has been introduced as a novel therapeutic option for dentin hypersensitivity. This technology enables sustained release and recharge of fluoride and other bioactive ions. It provides a durable polymeric seal, promotes dentinal tubule occlusion, and enhances biological activity at the tooth surface. These properties contribute to its desensitizing effect and potential for remineralization [7]. Clinical studies have demonstrated its effectiveness in providing both immediate and short-term hypersensitivity relief, showing comparable or superior performance to conventional fluoride varnishes [5, 7]. Furthermore, recent evidence highlights its clinical applicability in challenging oral environments because of its resistance to acidic dissolution and long-term stability of the protective polymeric barrier [8].

Bioactive glass powder is used through an air-polishing system as a bioactive prophylactic agent. In the oral environment, it releases calcium and phosphate ions and forms hydroxycarbonate apatite (HCA), a stable and acid-resistant mineral phase similar to natural tooth apatite. These mineral deposits seal dentinal tubules and provide protection. Continuous ion release supports long-term tubule occlusion and surface protection [1]. Randomized clinical trials have demonstrated significant reductions in dentin hypersensitivity following application of bioactive glass–based desensitizing systems, with favourable short- to medium-term clinical outcomes compared with conventional fluoride therapies [1, 9]. In addition, systematic reviews have reported that bioactive glass materials promote dentinal tubule occlusion and mineral deposition, supporting their potential desensitizing effect [10, 11].

Despite their promising bioactive properties, clinical evidence supporting the effectiveness of these agents in managing DH remains limited, particularly regarding long-term performance and durability of desensitization. Although clinical studies have demonstrated favourable desensitizing outcomes with these approaches [5, 9], most have reported only short-term follow-up, highlighting the need for well-designed long-term comparative clinical investigations to evaluate their effectiveness in dentin hypersensitivity management. 

Therefore, this randomized clinical trial compared the clinical effectiveness of an S-PRG–based resin varnish with that of a bioactive glass–based air-polishing system in the management of cervical dentin hypersensitivity. The study also assessed the effect of both materials on tooth color change over 12 months. Two null hypotheses were tested: (1) there would be no statistically significant difference between S-PRG–based varnish and bioactive glass–based air polishing in reducing dentin hypersensitivity over 12 months; and (2) there would be no statistically significant difference between the two interventions in their effect on tooth color over the same period.

## Materials and methods

### Trial design and setting

This study was designed as a randomized, two parallel-group, superiority clinical trial with blinded outcome assessment and statistical analysis conducted at the Conservative Dentistry Department, Faculty of Dentistry, Cairo University, Egypt. Participants were recruited between November and December 2024, and follow-up assessments were completed in January 2026. The trial was designed and reported in accordance with the Consolidated Standards of Reporting Trials (CONSORT) guidelines for randomized clinical trials.

### Ethical approval and trial registration

Ethical approval was obtained from the Research Ethics Committee of the Faculty of Dentistry, Cairo University (Approval ID: 21123). The study followed the Declaration of Helsinki for research involving human participants. The clinical trial was prospectively registered at ClinicalTrials.gov on July 31, 2023, under registration number NCT05981625. The full trial protocol and statistical analysis plan are available from the corresponding author upon reasonable request. No modifications, deviations, or post-commencement changes were made to the trial protocol or pre-specified outcomes after registration.

### Sample size calculation

Sample size estimation was based on a previous randomized clinical trial [1], which reported a standard deviation of 0.87 for dentin hypersensitivity scores. The calculation assumed a true mean difference of 1 unit in VAS scores between groups. A minimum of 13 teeth per group was required to achieve 80% power at a two-sided alpha level of 0.05. To compensate for potential dropouts, the sample size was increased by 30%, resulting in 17 participants per group and a total of 34 participants. The sample size calculation was performed using PS software version 3.1.2 (independent t-test model). Because only one eligible hypersensitive tooth was included per participant, the sample size calculation based on teeth corresponded directly to the number of participants.

### Eligibility criteria and participant recruitment

Eligible participants were adults aged 21–40 years with good general and oral health. They presented with a clinical diagnosis of cervical dentin hypersensitivity in anterior teeth and reported a VAS score ≥ 4 in response to evaporative and thermal stimuli. When multiple sensitive teeth were present, only one tooth per participant was selected to avoid clustering and ensure independent observations; therefore, the participant and tooth represented the same experimental unit. Exclusion criteria included teeth with mobility grade II or III, presence of active periodontal disease, caries, restorations, cracks, or pulpal pathology. Additional exclusions included use of anti-inflammatory or analgesic medications, history of allergy to study materials, xerostomia, smoking, pregnancy or lactation, or any medical condition affecting oral health or study compliance.

Comprehensive personal, medical, and dental histories were collected from all participants. Eligible individuals received detailed verbal and written information about the study’s objectives, procedures, duration, potential risks, and benefits. All participants provided written informed consent using a form approved by the Research Ethics Committee, confirming voluntary participation.

### Randomization, allocation concealment, and blinding

Simple randomization (1:1 ratio) was performed to assign participants to either the experimental group (S-PRG–based resin varnish) or the active comparator group (bioactive glass–based air-polishing system). The random allocation sequence was generated using an online random sequence generator (Randomness and Integrity Services Ltd; https://www.random.org) by an independent researcher who was not involved in participant recruitment, clinical procedures, or outcome assessments. To ensure strict allocation concealment and prevent selection bias, sequentially numbered, opaque, and sealed envelopes were prepared independently and stored in a centralized location by this researcher. Each envelope was sequentially opened by the principal investigator—who also conducted participant recruitment and enrolment—only after a participant’s eligibility was verified, formal registration was complete, and baseline assessments were obtained. The assigned allocation number was subsequently recorded in the participant’s chart to ensure complete traceability. Due to distinct differences in clinical application protocols and material presentation, complete participant and operator blinding was unfeasible; however, participants were informed that two separate desensitizing approaches were being evaluated. Crucially, the outcome assessors and the statistician remained fully blinded to group allocations throughout the entire study period.

### Interventions and materials

**Fig. 1:**
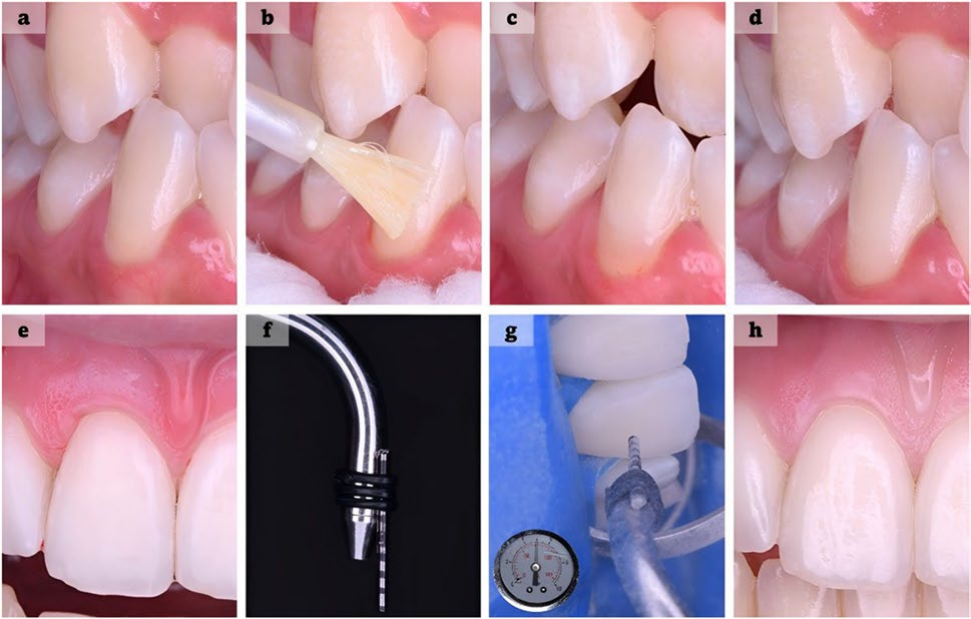
Representative clinical cases of both treatment groups. Panels (**a**–**d**) show the S-PRG–based varnish group: (**a**) preoperative view; (**b**) application; (**c**) immediate postoperative appearance with transient surface color alteration; and (**d**) 12-month follow-up showing resolution of the initial color change. Panels (**e**–**h**) show the bioactive glass–based air-polishing system group: (**e**) preoperative view; (**f**) standardized periodontal spacer for distance control; (**g**) application; and (**h**) 12-month follow-up

Two desensitizing systems were evaluated: (1) a surface pre-reacted glass-ionomer (S-PRG)–based resin system (light-cured resin coating material containing S-PRG fillers) and (2) a bioactive glass–based air-polishing system (calcium sodium phosphosilicate powder delivered via an air-polishing device). All materials were used according to the manufacturers’ instructions. Detailed material specifications, composition, and manufacturers are provided in Table 1. All operative procedures were performed by a single calibrated operator under standardized clinical conditions to ensure consistency of the interventions (Fig. 1).

**Table 1:** Materials’ trade name, specification, composition, and manufacturer

Material Trade name	Specification	Composition	Manufacturer
PRG Barrier Coat	Light-cured, S-PRG resin varnish	Base: S-PRG filler based on fluoroboro-aluminosilicate glass, Distilled water, Methacrylic acid monomer, and others. Active: Phosphonic acid monomer, Methacrylate monomer, Bis-MPEPP, Carboxylic acid monomer, TEGDMA, Reaction initiator, and others.	Shofu Inc., Kyoto, Japan
Sylc Original SR	Bioactive glass powder	45S5 Bioglass – calcium sodium phosphosilicate; elemental component: Silicon, Calcium, Sodium, Phosphorus, Oxygen.	Denfotex Research Ltd., London, United Kingdom

### Pre-operative standardization procedures

Participants received standardized oral hygiene and dietary instructions before the intervention [12]. Oral hygiene instructions included the modified Bass brushing technique and twice-daily brushing using a non-fluoridated toothpaste. Dietary instructions included avoidance of acidic foods and beverages throughout the study period [13]. Baseline prophylactic oral care was performed for all participants, including scaling and polishing with a fluoride-free paste, to standardize the oral environment before the intervention [14]. Participants were instructed not to use any additional desensitizing treatments or products during the study period.

### Assessment of dentin hypersensitivity

 Dentin hypersensitivity was evaluated using three standardized stimuli: tactile, evaporative, and thermal, combined with a 10-cm Visual Analog Scale (VAS) for pain assessment. Pain intensity categories were defined as: 0 = no pain; 1–3 = mild pain; 4–6 = moderate pain; and 7–10 = severe pain [15]. To improve scoring reliability and patient comprehension, laminated VAS cards displaying numerical scales, color coding, and facial expression illustrations were used. Before each assessment, adjacent teeth were isolated with Teflon tape to prevent unintended stimulation of neighboring teeth. Each stimulus was applied for 1–5 s, depending on patient response, with a mandatory 5-minute recovery interval between tests to avoid stimulus interaction and ensure pulp recovery. Participants independently recorded their pain scores following each stimulus application [16].

### Tactile stimulus

A sharp new dental explorer (Hu-Friedy Mfg. Co., LLC, Chicago, IL, USA) was gently moved across the exposed cervical dentin surface in an apico-coronal direction using standardized short strokes. The stimulus was applied uniformly to the central area of the exposed cervical dentin surface while avoiding contact with adjacent sound enamel [1].

### Evaporative stimulus

A standardized, continuous air blast was delivered using a dental air–water syringe under controlled conditions (air pressure ≈ 50 psi, temperature ≈ 20 °C). The syringe tip was positioned 3–4 mm from the exposed cervical dentin surface. To standardize this distance, the tip of a graduated periodontal probe was attached to the air syringe tip and used as a reference during stimulus application[17, 18].

### Thermal stimulus

A cold stimulus was applied using a 3-mm diameter cotton pellet sprayed with refrigerant spray (Endo Ice, Maquira, Maringá, PR, Brazil). The pellet was applied to the exposed cervical dentin surface [15, 19].

### Intervention protocols

#### S-PRG–based resin varnish application (experimental group)

Prior to application, the target tooth surface was cleaned using a prophylaxis polishing brush without paste (Nylon LATCH R.A. Prophylaxis Polishing Brushes, Dentamerica Inc., San Jose, CA, USA). Isolation was achieved using disposable cotton rolls (RISTEA, Shanghai Ristea Industries Co., Ltd., Shanghai, China), a plastic double-headed cheek retractor (Cotisen Cheek Retractor, Huanghua Promisee Dental Co., Ltd., Hebei, China) and a high-volume saliva ejector (ZT High Saliva Ejector, ZT Dental, Shanghai, China).

The base and active components of the S-PRG–based resin varnish were then mixed with the provided disposable applicator tip, following the manufacturer’s instructions. A thin uniform layer was applied to the exposed dentin surface and left undisturbed for 3 s, followed by light curing for 10 s using a calibrated LED curing unit (1200 mW/cm²)0.5 After polymerization, the oxygen-inhibited layer was gently removed using a moist cotton pellet [20].

#### Bioactive glass–based air-polishing system application (active comparator group)

Bioactive glass powder was delivered using an air-polishing device (ProJet 1, Voldent Medical Technology Co., Ltd., Guangxi, China) equipped with a 0.6-mm-diameter nozzle (particle size range: 38–90 μm). The device generates a pressurized air–water–powder slurry. Air pressure was standardized at 5 bar and verified using the dental unit’s built-in manometer immediately before each application.

Rubber dam isolation was used to prevent powder ingestion and soft tissue injury [16]. To standardize the working distance, a periodontal probe (Periodontal Probe 10 Marking Color Coded, SMIZ Dental, London, UK) was sectioned to approximately 4 mm and positioned adjacent to the nozzle as a spacer. The nozzle was held perpendicular to the tooth surface at a controlled distance of 3–4 mm.1 The powder was applied using gentle circular movements for 5–10 s, with continuous high-volume suction [5].

### Outcome assessment

Two experienced conservative dentistry clinicians (each with more than 15 years of clinical experience) were trained and calibrated in both Visual Analog Scale (VAS) pain assessment procedures and the modified United States Public Health Service (USPHS) evaluation criteria two weeks before study initiation. Inter-examiner agreement was assessed during calibration sessions using repeated independent evaluations, achieving a Cohen’s kappa coefficient of 0.87. Pain intensity, as the primary outcome measure, was assessed using the Visual Analog Scale (VAS). Tooth color change, as the secondary outcome, was assessed using the modified United States Public Health Service (USPHS) criteria with scores categorized as Alpha (no color mismatch), Bravo (slight but clinically acceptable mismatch), and Charlie (clinically unacceptable mismatch requiring intervention). Clinical evaluations were performed at baseline, immediately post-treatment, and at 1, 6, and 12 months post-intervention. VAS outcomes were analyzed both as continuous numerical values and as categorical severity levels to enhance both statistical sensitivity and clinical interpretability. Adverse events or unintended effects related to the interventions (such as mucosal irritation, allergic reactions, or postoperative discomfort) were monitored at each follow-up visit and recorded if present.

### Statistical analysis

Statistical analysis was performed using MedCalc software (version 22, MedCalc Software Ltd., Ostend, Belgium). Normality of continuous data was assessed using the Kolmogorov–Smirnov and Shapiro–Wilk tests. Parametric VAS data were expressed as mean ± standard deviation and analyzed using independent t-tests for intergroup comparisons (*P* ≤ 0.05) and repeated-measures ANOVA with Bonferroni correction for intragroup comparisons (*P* ≤ 0.005). For longitudinal categorical evaluation under an intention-to-treat (ITT) framework, continuous VAS scores were dichotomized into binary clinical outcomes (treatment success versus failure). Missing longitudinal categorical outcome data from participants lost to follow-up were conservatively handled via non-responder imputation, where dropouts were explicitly imputed as treatment failures in the data matrix and analyzed using Cochran’s Q test. Nonparametric categorical data were expressed as frequencies and percentages. Intergroup comparisons were conducted using the Mann–Whitney U test (*P* ≤ 0.05), whereas intragroup comparisons were performed using the Friedman test with appropriate multiple-comparison corrections (*P* ≤ 0.005 for VAS and *P* ≤ 0.008 for color change). The confidence limit was set at 95%, with a statistical power of 80%. All tests were two-tailed. Statistical analyses were conducted at the participant level, with one tooth representing one participant.

## Results

### Demographic and baseline characteristics

**Fig. 2:**
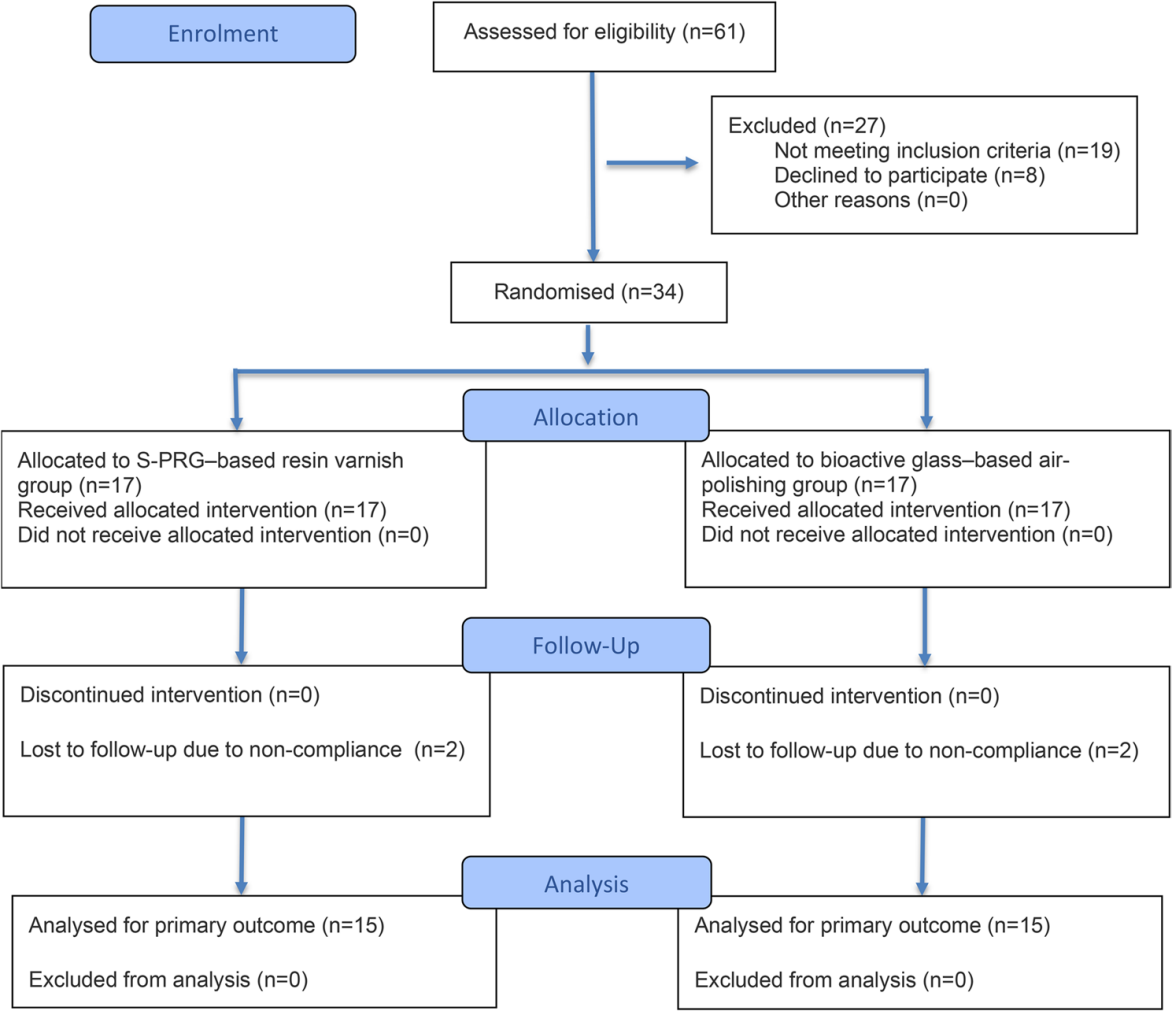
CONSORT flow diagram of participant enrolment, allocation, follow-up, and analysis

A total of 34 participants diagnosed with dentin hypersensitivity were enrolled and randomly allocated to the intervention and comparator groups (*n* = 17 each). At the 12-month follow-up, 30 participants completed the study, yielding an overall retention rate of 88.2%, indicating good compliance with the study protocol and follow-up (Fig. 2). The overall mean age of the study population was 22.46 ± 1.59 years, with no statistically significant difference between the two groups (*P* = 0.823). Baseline gender distribution was comparable between groups, with no significant intergroup difference (*P* = 0.718). In addition, the distribution of treated teeth between maxillary and mandibular anterior teeth did not differ significantly between the groups (*P* = 0.9216), confirming baseline intergroup homogeneity (Table 2). No adverse events or unintended effects related to either intervention were reported during the study period. 

**Table 2:** Baseline demographic and clinical characteristics of the study groups. Values are presented as mean ± standard deviation for continuous variables and number (percentage) for categorical variables

Variable	S-PRG–based resin varnish (*n* = 17)	Bioactive glass–based air polishing system (*n* = 17)	Total (*n* = 34)	*P* value
Age (years)	22.53 ± 1.68	22.40 ± 1.55	22.46 ± 1.59	0.8231
Gender, *n* (%)				
Male	5 (29.4%)	6 (35.3%)	11 (32.4%)	0.7180
Female	12 (70.6%)	11 (64.7%)	23 (67.6%)	0.7180
Tooth type, *n* (%)				
Maxillary central incisor	5 (29.4%)	6 (35.3%)	11 (32.4%)	0.9216
Maxillary lateral incisor	4 (23.5%)	3 (17.6%)	7 (20.6%)	0.9216
Maxillary canine	4 (23.5%)	5 (29.4%)	9 (26.5%)	0.9216
Mandibular canine	4 (23.5%)	3 (17.6%)	7 (20.6%)	0.9216

### Primary outcome (VAS pain intensity)

#### Tactile stimulus

Baseline tactile pain scores were mild and comparable between groups (*P* = 1.000). No significant intergroup differences were observed immediately after treatment or at the 1- and 6-month follow-up visits (*P* > 0.05). At 12 months, the S-PRG–based resin varnish maintained low pain scores (1.27 ± 0.46), whereas the bioactive glass–based air polishing group demonstrated a marked increase in pain intensity (2.73 ± 1.03), resulting in a statistically significant intergroup difference (*P* < 0.0001). The mean intergroup difference at 12 months was − 1.47 VAS units (95% CI: −2.08 to − 0.86), favouring the S-PRG–based resin varnish group. Intragroup analysis revealed a sustained and significant reduction in tactile pain in the S-PRG–based resin varnish group across all follow-up intervals, whereas the bioactive glass–based air polishing group demonstrated an initial reduction in pain through the 6-month follow-up, followed by a significant late relapse at 12 months (*P* < 0.001) (Table 3).

#### Evaporative stimulus

Baseline evaporative pain scores were severe and comparable between groups (*P* = 0.8228). Immediately after treatment, significantly lower pain scores were recorded in the S-PRG–based resin varnish group compared with the bioactive glass–based air polishing group (*P* = 0.0023). This significant intergroup difference persisted at 1 month (*P* = 0.0376), 6 months (*P* < 0.0001), and 12 months (*P* < 0.0001). At the 12-month follow-up, the mean intergroup difference was − 6.0 VAS units (95% CI: −6.56 to − 5.44), favouring the S-PRG–based resin varnish group. Intragroup analysis revealed a sustained and significant reduction in evaporative pain in the S-PRG–based resin varnish group across all follow-up intervals, whereas the bioactive glass–based air polishing group demonstrated an initial reduction in pain through the 6-month follow-up, followed by a significant late relapse at 12 months (*P* < 0.001) (Table 3).

#### Thermal stimulus

Baseline thermal pain scores were severe and comparable between groups (*P* = 0.1272). Immediately post-treatment, significantly lower pain scores were observed in the S-PRG–based resin varnish group compared with the bioactive glass–based air polishing group (*P* = 0.0052). This intergroup difference remained significant at 1 month (*P* = 0.0401), 6 months (*P* = 0.0009), and 12 months (*P* < 0.0001). At the 12-month follow-up, the mean intergroup difference was − 5.93 VAS units (95% CI: −6.63 to − 5.23), favouring the S-PRG–based resin varnish group. Intragroup analysis revealed a sustained and significant reduction in thermal pain in the S-PRG–based resin varnish group across all follow-up intervals, whereas the bioactive glass–based air polishing group demonstrated an initial reduction in pain through the 6-month follow-up, followed by a significant late relapse at 12 months (*P* < 0.001) (Table 3). 

**Table 3:** Mean ± standard deviation (SD) of Visual Analog Scale (VAS) pain scores for tactile, evaporative, and thermal stimuli across follow-up periods

Stimulus	Follow-up	S-PRG–based resin varnish		Bioactive glass–based air polishing system		*P* value
		Rank	Mean ± SD	Rank	Mean ± SD	
Tactile stimulus	Baseline	a	2.80 ± 0.68	a	2.80 ± 0.68	1.0000
	Immediate	b	2.27 ± 0.46	a	2.53 ± 0.74	0.2467
	1 month	c	1.40 ± 0.63	b	1.60 ± 0.51	0.3475
	6 months	c	1.33 ± 0.49	b	1.60 ± 0.51	0.1534
	12 months	c	1.27 ± 0.46	a	2.73 ± 1.03	< 0.0001*
	*P* value	< 0.001*		< 0.001*		
Evaporative stimulus	Baseline	a	7.73 ± 0.88	a	7.67 ± 0.72	0.8228
	Immediate	b	4.73 ± 0.96	b	5.67 ± 0.49	0.0023*
	1 month	c	2.07 ± 0.80	c	2.60 ± 0.51	0.0376*
	6 months	d	1.53 ± 0.64	c	2.73 ± 0.46	< 0.0001*
	12 months	d	1.40 ± 0.51	a	7.40 ± 0.91	< 0.0001*
	*P* value	< 0.001*		< 0.001*		
Thermal stimulus	Baseline	a	8.00 ± 0.65	a	8.40 ± 0.74	0.1272
	Immediate	b	4.93 ± 0.80	b	5.67 ± 0.49	0.0052*
	1 month	c	2.13 ± 0.99	c	2.80 ± 0.68	0.0401*
	6 months	d	1.60 ± 0.74	c	2.60 ± 0.74	0.0009*
	12 months	d	1.53 ± 0.52	a	7.47 ± 1.19	< 0.0001*
	*P* value	< 0.001*		< 0.001*		

*Statistically significant difference (*P* ≤ 0.05). Different rank letters within the same group indicate statistically significant differences between follow-up periods (*P* ≤ 0.05)

#### Categorical VAS pain analysis

Pain responses to tactile, evaporative, and thermal stimuli were assessed at baseline, immediately after treatment, and at 1, 6, and 12 months in both groups. Responses were categorized as no pain, mild, moderate, or severe. For tactile stimulation, both groups showed comparable pain distributions at baseline, with 88.2% reporting mild pain and 11.8% reporting moderate pain (*P* = 1.000). At 12 months, the S-PRG–based resin varnish group maintained a stable response, with 100% of cases reporting mild pain. In contrast, the bioactive glass–based air polishing group demonstrated deterioration, with 73.3% reporting mild pain and 26.7% reporting moderate pain, resulting in a statistically significant intergroup difference (*P* = 0.0347). Intragroup analysis demonstrated no significant changes across follow-up in either group; however, the bioactive glass–based air polishing group showed a pattern suggestive of partial relapse at the 12-month follow-up (Table 4).

For evaporative stimulation, both groups exhibited severe pain in 100% of cases at baseline (*P* = 1.000). The S-PRG–based resin varnish group showed progressive improvement, reaching 100% mild pain by 1 month, which was maintained at all subsequent evaluation points. Conversely, the bioactive glass–based air polishing group demonstrated initial improvement through the 6-month follow-up, followed by marked relapse at 12 months, with 86.7% reporting severe pain and 13.3% reporting moderate pain, yielding a highly statistically significant intergroup difference (*P* < 0.0001). Intragroup analysis confirmed sustained improvement in the S-PRG–based resin varnish group and significant relapse in the bioactive glass–based air polishing group (Table 4).

For thermal stimulation, both groups reported severe pain in 100% of cases at baseline (*P* = 1.000). The S-PRG–based resin varnish group showed progressive improvement, achieving 100% mild pain by 6 months, which remained unchanged at the 12 month follow-up. In contrast, the bioactive glass–based air polishing group demonstrated initial improvement through the 6-month follow-up, followed by marked relapse at 12 months, with 73.3% reporting severe pain and 26.7% reporting moderate pain, resulting in a significant intergroup difference (*P* < 0.0001). Intragroup analysis demonstrated sustained improvement in the S-PRG–based resin varnish group, whereas the bioactive glass-based air polishing group exhibited significant deterioration at the 12-month follow-up (Table 4). 

**Table 4:** Frequency and percentage distribution of categorical VAS pain responses for tactile, evaporative, and thermal stimuli across follow-up periods

Stimulus	Follow-up	S-PRG–based resin varnish					Bioactive glass–based air polishing system					*P* value
		Rank	No pain	Mild	Moderate	Severe	Rank	No pain	Mild	Moderate	Severe	
Tactile stimulus	Baseline	a	0(0%)	15(88.2%)	2(11.8%)	0(0%)	a	0(0%)	15(88.2%)	2(11.8%)	0(0%)	1.0000
	Immediate	a	0(0%)	15(100%)	0(0%)	0(0%)	a	0(0%)	13(86.7%)	2(13.3%)	0(0%)	0.1501
	1 month	a	0(0%)	15(100%)	0(0%)	0(0%)	a	0(0%)	15(100%)	0(0%)	0(0%)	1.0000
	6 months	a	0(0%)	15(100%)	0(0%)	0(0%)	a	0(0%)	15(100%)	0(0%)	0(0%)	1.0000
	12 months	a	0(0%)	15(100%)	0(0%)	0(0%)	a	0(0%)	11(73.3%)	4(26.7%)	0(0%)	0.0347*
	*P* value	0.0861					0.0249					
Evaporative stimulus	Baseline	a	0(0%)	0(0%)	0(0%)	17(100%)	a	0(0%)	0(0%)	0(0%)	17(100%)	1.0000
	Immediate	b	0(0%)	2(13.3%)	13(86.7%)	0(0%)	c	0(0%)	0(0%)	15(100%)	0(0%)	0.1501
	1 month	c	0(0%)	15(100%)	0(0%)	0(0%)	d	0(0%)	15(100%)	0(0%)	0(0%)	1.0000
	6 months	c	0(0%)	15(100%)	0(0%)	0(0%)	d	0(0%)	15(100%)	0(0%)	0(0%)	1.0000
	12 months	c	0(0%)	15(100%)	0(0%)	0(0%)	b	0(0%)	0(0%)	2(13.3%)	13(86.7%)	< 0.0001*
	*P* value	< 0.00001*					< 0.00001*					
Thermal stimulus	Baseline	a	0(0%)	0(0%)	0(0%)	17(100%)	a	0(0%)	0(0%)	0(0%)	17(100%)	1.0000
	Immediate	b	0(0%)	0(0%)	15(100%)	0(0%)	b	0(0%)	0(0%)	15(100%)	0(0%)	1.0000
	1 month	c	0(0%)	13(86.7%)	2(13.3%)	0(0%)	d	0(0%)	14(93.3%)	1(6.7%)	0(0%)	0.5496
	6 months	d	0(0%)	15(100%)	0(0%)	0(0%)	d	0(0%)	15(100%)	0(0%)	0(0%)	1.0000
	12 months	d	0(0%)	15(100%)	0(0%)	0(0%)	c	0(0%)	0(0%)	4(26.7%)	11(73.3%)	< 0.0001*
	*P* value	< 0.00001*					< 0.00001*					

*Statistically significant intergroup differences were considered at *P* ≤ 0.05. Different rank letters within the same group indicate statistically significant pairwise differences between follow-up periods (*P* ≤ 0.005)

### Secondary outcome (tooth color change)

Immediately after treatment, the S-PRG–based resin varnish group demonstrated 100% Bravo ratings, whereas the bioactive glass–based air polishing group demonstrated 100% Alpha ratings, resulting in a statistically significant intergroup difference (*P* < 0.0001). At the 1-, 6-, and 12-month follow-up visits, both groups exhibited 100% Alpha ratings, with no significant intergroup differences (*P* = 1.000). Longitudinal analysis revealed a significant improvement in tooth color over time in the S-PRG-based resin varnish group. In contrast, the bioactive glass-based air polishing group maintained stable Alpha ratings across all follow-up intervals, indicating superior immediate esthetic performance (Table 5).

**Table 5:** Frequency and percentage of color change scores, showing intergroup comparisons between the two groups at each follow-up interval, and intragroup comparisons across different follow-up periods

Follow-up	S-PRG–based resin varnish				Bioactive glass–based air polishing system				*P* value
	Rank	Alpha	Bravo	Charlie	Rank	Alpha	Bravo	Charlie	
Immediate	a	0(0%)	17(100%)	0(0%)	a	17(100%)	0(0%)	0(0%)	< 0.0001*
1 month	b	15(100%)	0(0%)	0(0%)	a	15(100%)	0(0%)	0(0%)	1.0000
6 months	b	15(100%)	0(0%)	0(0%)	a	15(100%)	0(0%)	0(0%)	1.0000
12 months	b	15(100%)	0(0%)	0(0%)	a	15(100%)	0(0%)	0(0%)	1.0000
*P* value	< 0.0001*				1.0000				

*Statistically significant difference (*P*≤ 0.05). Different rank letters within the same group indicate statistically significant differences between follow-up periods

## Discussion

The clinical findings of this randomized trial demonstrate that both the S-PRG–based resin varnish and the bioactive glass–based air-polishing system successfully reduce dentin hypersensitivity, with distinct profiles regarding immediate versus sustained therapeutic relief. The S-PRG–based varnish demonstrated significantly superior and more sustained pain reduction across tactile, evaporative, and thermal stimuli throughout the 12-month follow-up. Accordingly, the first null hypothesis was rejected.

The superior long-term performance of the S-PRG varnish can be attributed to its combined physicochemical and bioactive mechanisms [21]. S-PRG fillers release multiple ions, including fluoride, strontium, sodium, borate, aluminum, and silicate, that contribute to dentinal tubule occlusion, surface remineralization, and chemical stabilization of dentin [22, 23]. Fluoride and strontium ions form acid-resistant fluorapatite and strontium-apatite phases, enhancing resistance to acid dissolution and promoting remineralization of the peritubular dentin substrate [8]. Borate ions increase local pH, buffer acidic challenges, and further stabilize the occlusive interface by reducing fluid flow within dentinal tubules [21, 24]. In vitro studies additionally suggest that S-PRG eluates inhibit bacterial adhesion and biofilm formation on dentin surfaces, potentially minimizing microbial-mediated acid attack and preserving occlusion integrity over time [25–27]. Collectively, these synergistic effects, including tubule sealing, ion-mediated remineralization, acid buffering, and antimicrobial activity, may explain the sustained reduction in dentin hypersensitivity observed with S-PRG–based varnish. Previous studies similarly support the immediate and long-term desensitizing potential of S-PRG–containing materials [27, 28].

In contrast, bioactive glass particles primarily exert their desensitizing effect through rapid ion exchange and hydroxycarbonate apatite deposition on the dentin surface and within tubules. While this layer provides immediate symptom relief, evidence indicates it is often superficial and loosely adherent, making it susceptible to dissolution by saliva, dietary acids, and mechanical disruption from oral hygiene practices [1]. Over time, this superficial nature may permit tubule re-exposure and fluid movement, leading to recurrence of sensitivity symptoms [28, 29]. Systematic reviews indicate that bioactive glass desensitizers are effective in reducing pain in the short- to medium-term (4–12 weeks), but long-term stability remains variable [11, 29, 30]. These findings provide a rational explanation for the late relapse in pain scores observed at the 12-month follow-up in the bioactive glass air polishing group. Nevertheless, bioactive glass-based systems may provide effective short-term symptom relief and could be considered in cases where immediate but temporary desensitization is clinically sufficient [9].

While in vitro studies consistently show dentinal tubule occlusion with bioactive glass and hydroxyapatite materials, these controlled conditions, lacking continuous acid challenges, saliva flow, and mechanical wear, do not fully mimic the oral environment and may overestimate the durability of the occlusive layer formed under these conditions compared with in vivo conditions [11]. Additionally, a recent systematic review revealed that bioactive glass composition (calcium sodium phosphosilicate versus fluoride-containing variants) and particle size influence ion release kinetics and mineral precipitation. Conventional calcium sodium phosphosilicate formulations showed no significant difference compared with positive controls, whereas newer fluoro calcium phosphosilicate variants performed better at managing dentin hypersensitivity and appear to achieve greater long-term symptom reduction [22, 31].

The present findings align with previous studies reporting that resin-based desensitizing systems incorporating S-PRG fillers demonstrate superior maintenance of tubule occlusion, enhanced resistance to acidic dissolution, and prolonged ion-mediated remineralization potential, supporting their clinical longevity [28, 32, 33]. The current results therefore provide clinical evidence reinforcing the sustained effectiveness of S-PRG-based varnish in managing dentin hypersensitivity.

With respect to esthetic outcomes, the immediate and transient inferior tooth color change observed after S-PRG varnish application is most likely attributable to the surface optical properties of the resin coating rather than to intrinsic discoloration. The thin polymer film introduces refractive-index differences relative to the underlying tooth structure, and variations in film thickness and surface texture may further modify light reflection and scattering [34]. These optical effects are most pronounced immediately after application and gradually diminish as the resin layer equilibrates within saliva and undergoes mechanical wear [35]. This is consistent with the study’s clinical observation of an initially inferior color match that improved progressively over subsequent follow-up intervals. Accordingly, the second null hypothesis was also rejected.

Overall, the results of this study have important implications for evidence-based clinical decision-making in the management of dentin hypersensitivity. The sustained efficacy of the S-PRG-based varnish suggests suitability for long-term symptom control, whereas bioactive glass systems may serve as effective short-term interventions but appear more susceptible to degradation under oral challenges. Despite the strengths of this trial, several limitations should be acknowledged. The relatively modest sample size and single-center design may limit generalizability of the findings. Although a 12-month follow-up is clinically meaningful, longer observation periods are necessary to fully characterize material durability and long-term stability of dentinal tubule occlusion. Assessment of dentin hypersensitivity relied partly on patient-reported outcome measures, while tooth color evaluation was based on visual clinical assessment rather than instrumental spectrophotometric analysis, introducing an inherent degree of subjectivity. Complete participant and operator blinding was not feasible because of differences in intervention protocols and material presentation. Furthermore, the absence of repeated application protocols precludes assessment of potential cumulative effects over time. The relatively young study population may also limit extrapolation of the findings to older populations with different dentin characteristics and oral conditions.

Future research should explore long-term performance across more diverse populations, investigate the ultrastructural stability of tubule occlusion, ion diffusion kinetics, and surface bioactivity, and assess combined or adjunctive therapeutic strategies for optimizing long-term management of dentin hypersensitivity.

## Conclusion

Within the limitations of this study, the S-PRG–based resin varnish demonstrated a more sustained reduction in dentin hypersensitivity compared with the bioactive glass–based air-polishing system over the 12-month follow-up period. These findings suggest that S-PRG–based varnish may represent a promising option for the moderate-term management of dentin hypersensitivity.

## Data Availability

The data generated and/or analyzed during this study are available from the corresponding author upon reasonable request.
